# Let-7b inhibits cancer-promoting effects of breast cancer-associated fibroblasts through IL-8 repression

**DOI:** 10.18632/oncotarget.24895

**Published:** 2018-04-03

**Authors:** Bothina Al-Harbi, Siti-Fauziah Hendrayani, Gabriela Silva, Abdelilah Aboussekhra

**Affiliations:** ^1^ Department of Molecular Oncology, King Faisal Specialist Hospital and Research Center, Riyadh, Saudi Arabia; ^2^ Current/Present address: Instituto de Biologia Experimental e Tecnológica, Oeiras, Portugal

**Keywords:** breast cancer, let-7b, cancer-associated fibroblasts, IL-8

## Abstract

Cancer-associated fibroblasts (CAFs) are major players in the development and spread of breast carcinomas through non-cell-autonomous signaling. These paracrine effects are under the control of several genes and microRNAs. We present here clear evidence that let-7b, a tumor suppressor microRNA, plays key roles in the persistent activation of breast stromal fibroblasts and their functional interplay with cancer cells. We have first shown that let-7b is down-regulated in CAFs as compared to their corresponding normal adjacent fibroblasts, and transient specific let-7b inhibition permanently activated breast fibroblasts through induction of the IL-6-related positive feedback loop. More importantly, let-7b-deficient cells promoted the epithelial-to-mesenchymal transition process in breast cancer cells in an IL-8-dependent manner, and also enhanced orthotopic tumor growth *in vivo*. On the other hand, overexpression of let-7b by mimic permanently suppressed breast myofibroblasts through blocking the positive feedback loop, which inhibited their paracrine pro-carcinogenic effects. Furthermore, we have shown that let-7b negatively controls IL-8, which showed higher expression in the majority of CAF cells as compared to their adjacent normal counterparts, indicating that IL-8 plays a major role in the carcinoma/stroma cross-talk. These findings support targeting active stromal fibroblasts through restoration of let-7b/IL-8 expression as a therapeutic option for breast carcinomas.

## INTRODUCTION

Despite early detection and targeted therapies, breast cancer remains the most commonly diagnosed cancer and the leading cause of cancer death among women worldwide [1]. Breast carcinomas are composed of tumor cells and different types of non-carcinogenic cells that constitute tumor stroma. It became clear that the carcinoma-stroma interplay is critical for carcinogenesis through intercellular cross-talk. Fibroblasts, the most common and the most active type of stromal cells, are known as reactive fibroblasts, myofibroblasts or cancer-associated fibroblasts. Myofibroblasts have a prominent role in the growth, progression and spread of tumor cells through active release of various growth factors, cytokines and chemokines such as TGF-β1, SDF-1 and IL-6 [2, 3]. The active status of myofibroblasts is sustained even in absence of the activating signal, due mainly to the activation of the IL-6/STAT3/NF-κB positive feedback loop that links inflammation to cancer [4]. One of the important members of this loop is the Let-7b, which belongs to the let-7 family of microRNAs. Members of this family are highly conserved in sequence and function from *C. elegans* to humans, and play important roles in several physiological and pathological processes such as development, differentiation as well as carcinogenesis [5]. Functionally, let-7 family members act as tumor suppressor in several types of malignant human tumors by inhibiting the expression of oncogenes and key regulators of mitogenic pathways [6, 7]. Indeed, the expression of let-7 family members has been found reduced in many types of cancers [8]. Furthermore, it has been shown that the restoration of let-7b expression effectively inhibited the growth of lung and breast cancer cells *in vitro*, as well as in mouse models of hepatocellular carcinoma [6, 9, 10]. Importantly, it has been recently reported that let-7b expression was reduced in breast cancer tissues and was inversely associated with tumor lymph node metastasis, patient overall survival and relapse-free survival. Furthermore, breast cancer patients with low let-7b expression had poor prognoses, indicating that let-7b could act as tumor suppressor miRNA in breast cancer onset and spread [5, 11].

In the present report, we have shown that let-7b suppresses the pro-carcinogenic effects of breast cancer-associated fibroblasts through negative regulation of IL-8.

## RESULTS

### Breast cancer-associated fibroblasts express low level of let-7b, and specific inhibition of let-7b activates breast stromal fibroblasts

**Figure 1:**
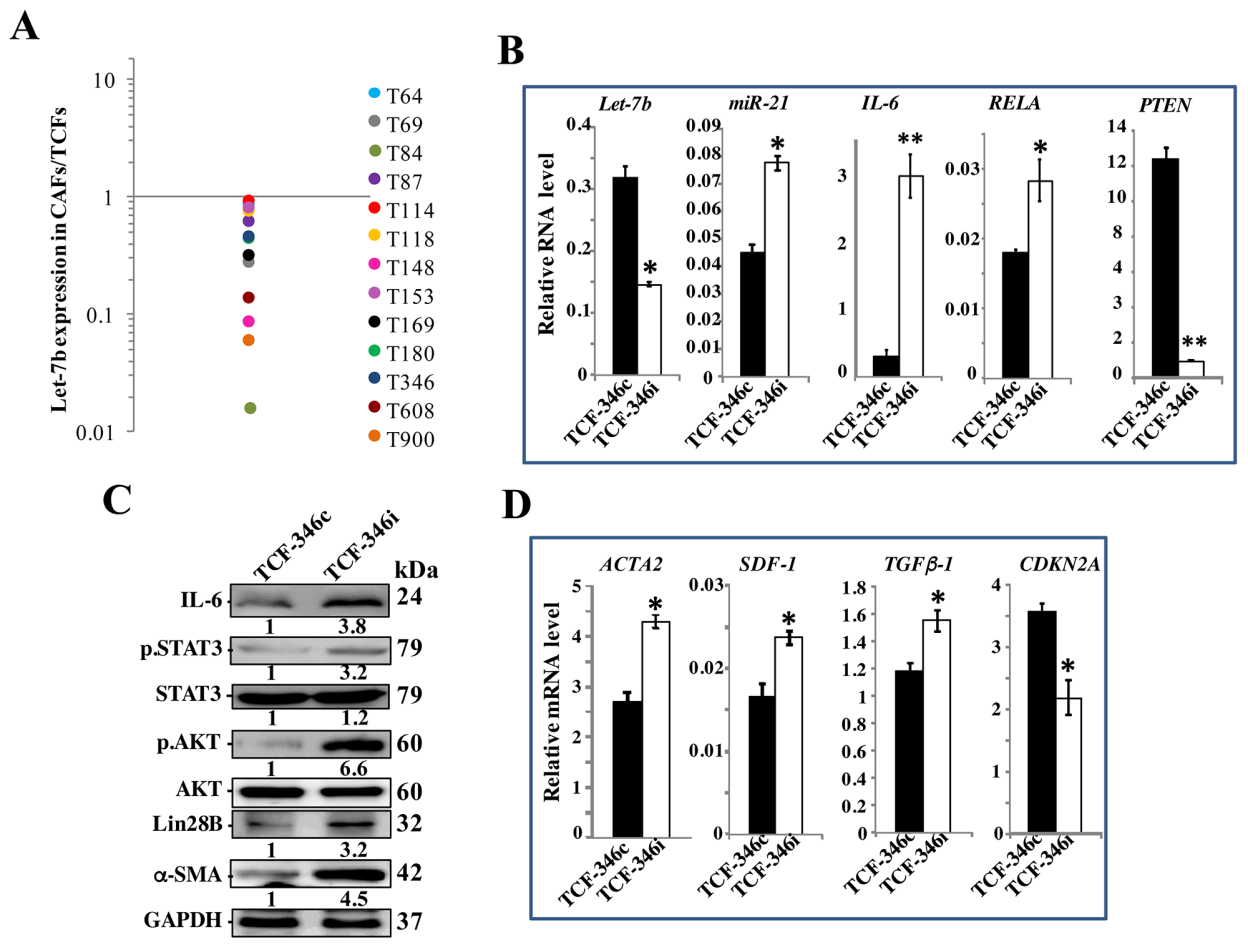
Let-7b inhibition activates breast stromal fibroblasts through the STAT3/NF-κB/let-7b positive feedback loop **(A)** Total RNA was purified from the indicated 13 CAF/TCF pairs (T64 to T900), and the level of let-7b was assessed by qRT-PCR. The values obtained in CAFs were divided by the values of their corresponding TCFs, and then were plotted. **(B** and **D)** TCF-346 cells were transfected with let-7b inhibitor (TCF-346i) or control (TCF-346c) for 48 h, and then medium was replaced with inhibitor-free medium for 24 h. Total RNA was purified and used for qRT-PCR. Experiments were performed in triplicate; error bars represent means ± S.D, values of 3 independent experiments (^*^P < 0.05 and ^**^P<0.001). **(C)** Whole cell lysates were prepared from the indicated cells, and were used for immunoblotting analysis using antibodies against the indicated proteins, and GAPDH was utilized as internal control. The numbers under the bands represent fold changes relative to GAPDH. For the phosphorylated proteins, the levels were determined relative to their respective non-phosphorylated forms.

To investigate the importance of let-7b in breast stromal fibroblasts (BSF) activation and the carcinoma-stroma functional interplay, we first assessed the level of this microRNA in 13 CAFs and their corresponding tumor counterpart fibroblasts (TCFs) derived from the same patients and cultured simultaneously. Total RNA was purified from these cells and quantitative RT-PCR (qRT-PCR) was utilized to amplify/quantify the let-7b microRNA. Figure 1A shows let-7b down-regulation in all CAFs relative to their corresponding TCFs, with great difference between the different pairs. This prompted us to test the possible implication of let-7b down-regulation in the activation of breast stromal fibroblasts. Therefore, normal breast stromal fibroblasts (TCF-346) were transfected with let-7b inhibitor (TCF-346i) or control sequence (TCF-346c) for 24 h, and then the levels of let-7b and its downstream targets were assessed by qRT-PCR. Figure 1B shows that let-7b level was significantly reduced, with concomitant strong up-regulation of *IL-6* (12 fold) in TCF-346i as compared to control cells. Similarly, let-7b inhibition increased the levels of miR-21 and *RELA* that codes for *NF-*κ*B* (p65), while it reduced the level of *PTEN* as compared to the control cells (Figure 1B). These results suggest that let-7b inhibition activated the IL-6-related positive feedback loop. To confirm this observation, we tested the effect of let-7b inhibition on the expression of Lin28B and the activation of STAT3 and AKT, three major components of the loop. To this end, cell lysates were prepared from TCF-346c and TCF-346i cells, and proteins were used for immunoblotting analysis. We first confirmed that let-7b inhibition increased the expression of IL-6 at the protein level as well (Figure 1C). Subsequently, we have shown that let-7b inhibition activated STAT3 (p-STAT3) and AKT (p-AKT), and up-regulated Lin28B, an inhibitor of let-7b (Figure 1C). This indicates that the inhibition of let-7b function activated the IL-6-dependent positive feedback loop in BSFs. Thereby, we tested the effect of let-7b inhibition on the activation of BSFs by assessing the levels of the three major markers of active fibroblasts (ACTA2 (α-SMA), SDF-1 and TGF-β1). Interestingly, let-7b inhibitor increased the mRNA levels of these three genes as compared to the negative control (Figure 1D). On the other hand, the level of the tumor suppressor gene *CDKN2A* was decreased in TCF-346i as compared to controls (Figure 1D). Similar results were obtained when TCF-346c and TCF-346i cells were passaged once and were reincubated in let-7b inhibitor-free medium (Supplementary Figure 1). These results suggest that let-7b inhibition activates breast stromal fibroblasts.

### Let-7b inhibition stably activates breast stromal fibroblasts

**Figure 2:**
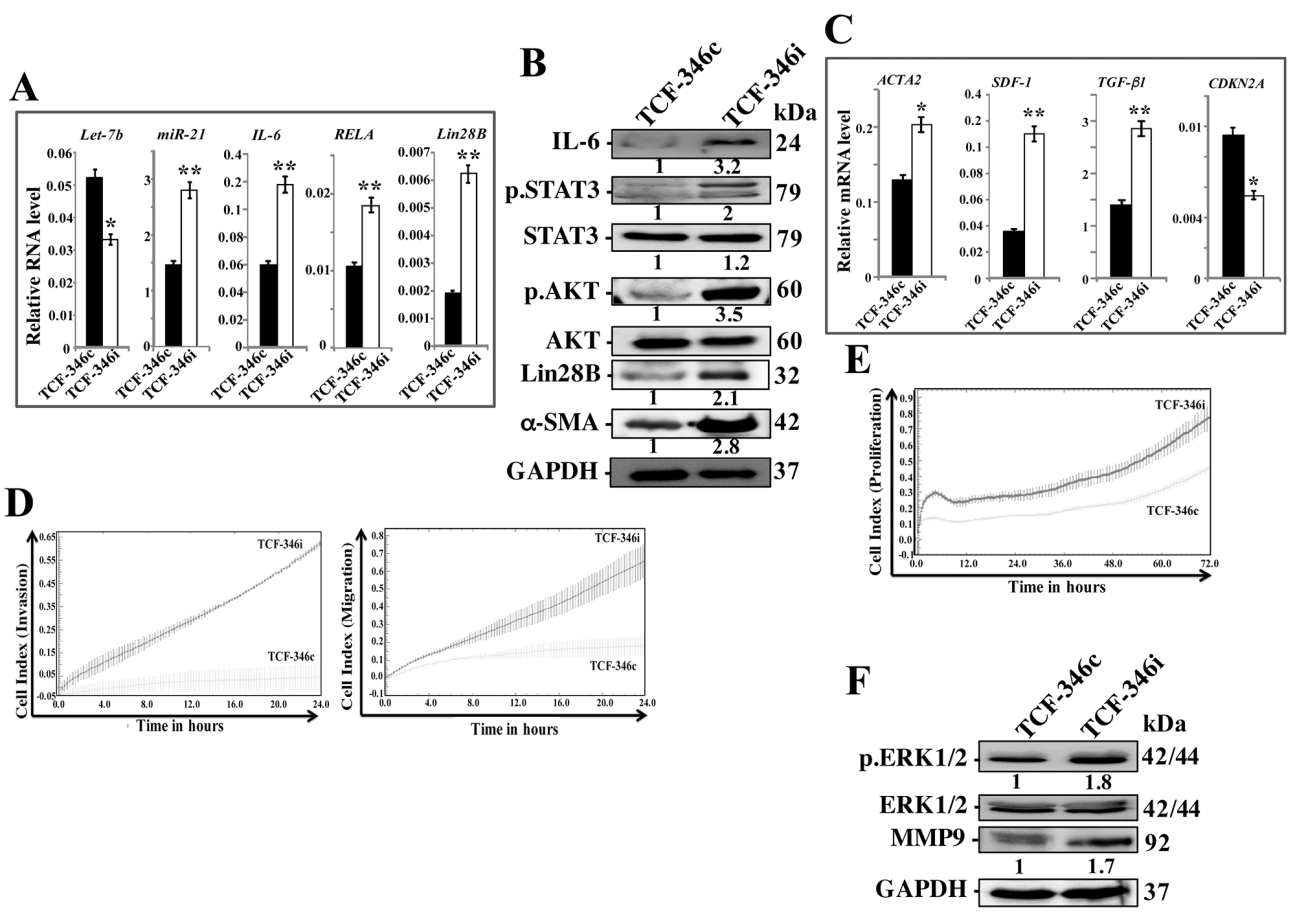
Let-7b inhibition persistently activates STAT3/NF-κB/let-7b positive feedback loop and enhances the invasion/migration/proliferation abilities of breast stromal fibroblasts TCF-346 cells were transfected with let-7b inhibitor (TCF-346i) or control (TCF-346c) for 48 h, and then the medium-containing let-7b inhibitor was removed and replaced with let-7b inhibitor-free medium for 24 h, and then cells were passaged and re-incubated in inhibitor-free medium 4 times. **(A** and **C)** Total RNA was purified from the indicated cells and used for qRT-PCR. Experiments were performed in triplicate; error bars represent means ± S.D, values of 3 independent experiments (^*^P < 0.05 and ^**^P<0.001). **(B** and **F)** Whole cell lysates were prepared from the indicated cells and used for immunoblotting analysis using antibodies against the indicated proteins. The numbers under the bands represent fold changes relative to GAPDH. For the phosphorylated proteins, the levels were determined relative to their respective non-phosphorylated forms. **(D** and **E)** Exponentially growing TCF-346c and TCF-346i cells (10^4^) in let-7b inhibitor-free medium were added independently into the upper chambers of the 16-well CIM-plates (invasion/migration), while the bottom chambers contained complete medium. For proliferation, cells were seeded in the E-plates in the presence of complete medium. Cell invasion, migration and proliferation were assessed using the RTCA-DP xCELLigence System. Data are representative of different experiments performed in triplicate.

We then asked whether let-7b inhibition-dependent activation of the loop and BSFs is permanent or transient. Therefore, the medium-containing let-7b inhibitor was removed and replaced with let-7b inhibitor-free medium for 48 h, and then cells were passaged and re-incubated in inhibition-free medium (IFM) 4 times. Figure 2A shows that *let-7b* remained down-regulated while the levels of miR-21, IL-6, *RELA* and Lin28B remained up-regulated in TCF-346i relative to controls, despite the removal of the let-7b inhibitor and cells were passaged several times. These findings were confirmed in other cells (TCF-64) (Supplementary Figure 2A), and at the protein level for IL-6 and Lin28B (Figure 2B). Furthermore, let-7b inhibition activated STAT3 (p-STAT3) and AKT (p-AKT) (Figure 2B). These results indicate that the transient let-7b inhibition activates the positive feedback loop in a persistent manner. Therefore, we also confirmed the persistent activation of BSFs upon let-7b inhibition by showing sustained up-regulation of the α-SMA protein (Figure 2B) and mRNA (Figure 2C) in TCF-346i relative to TCF-346c. Likewise, the levels of the SDF-1 and TGF β -1 mRNAs also remained higher in TCF-346i and TCF-64i relative to their respective controls (Figure 2C and Supplementary Figure 2B, respectively). On the other hand, the *CDKN2A* gene remained repressed (Figure 2C). This suggests that let-7b inhibition persistently transformed breast stromal fibroblasts to myofibroblasts likely through the activation of the IL-6-dependent positive feedback loop. To confirm this, we decided to investigate the effect of let-7b inhibition on the invasion/migration abilities of breast stromal fibroblasts. Therefore, exponentially growing TCF-346c and TCF-346i cells, in IFM and after many passaging, were seeded on the upper chamber wells of the CIM plates 16 with serum-free medium (SFM), while lower chamber wells contained complete medium (CpM). The invasion and migration of cells were assessed using the RTCA-DP xCELLigence System. Figure 2E shows increase in the invasion/migration abilities of TCF-346i cells as compared to TCF-346c cells. Using the same system, we have also shown that the proliferation rate of TCF-346i cells was higher than control cells (Figure 2E). This indicates that let-7b inhibition persistently increased the invasion/migration and proliferation abilities of breast stromal fibroblasts, which confirms their sustained activation. This was confirmed at the molecular level by showing the persistent activation of ERK1/2 and the up-regulation of MMP9 in TCF-346i cells as compared to controls (Figure 2F).

### Let-7b inhibition in breast fibroblasts triggers EMT in breast cancer cells in an IL-8-dependent paracrine fashion

**Figure 3:**
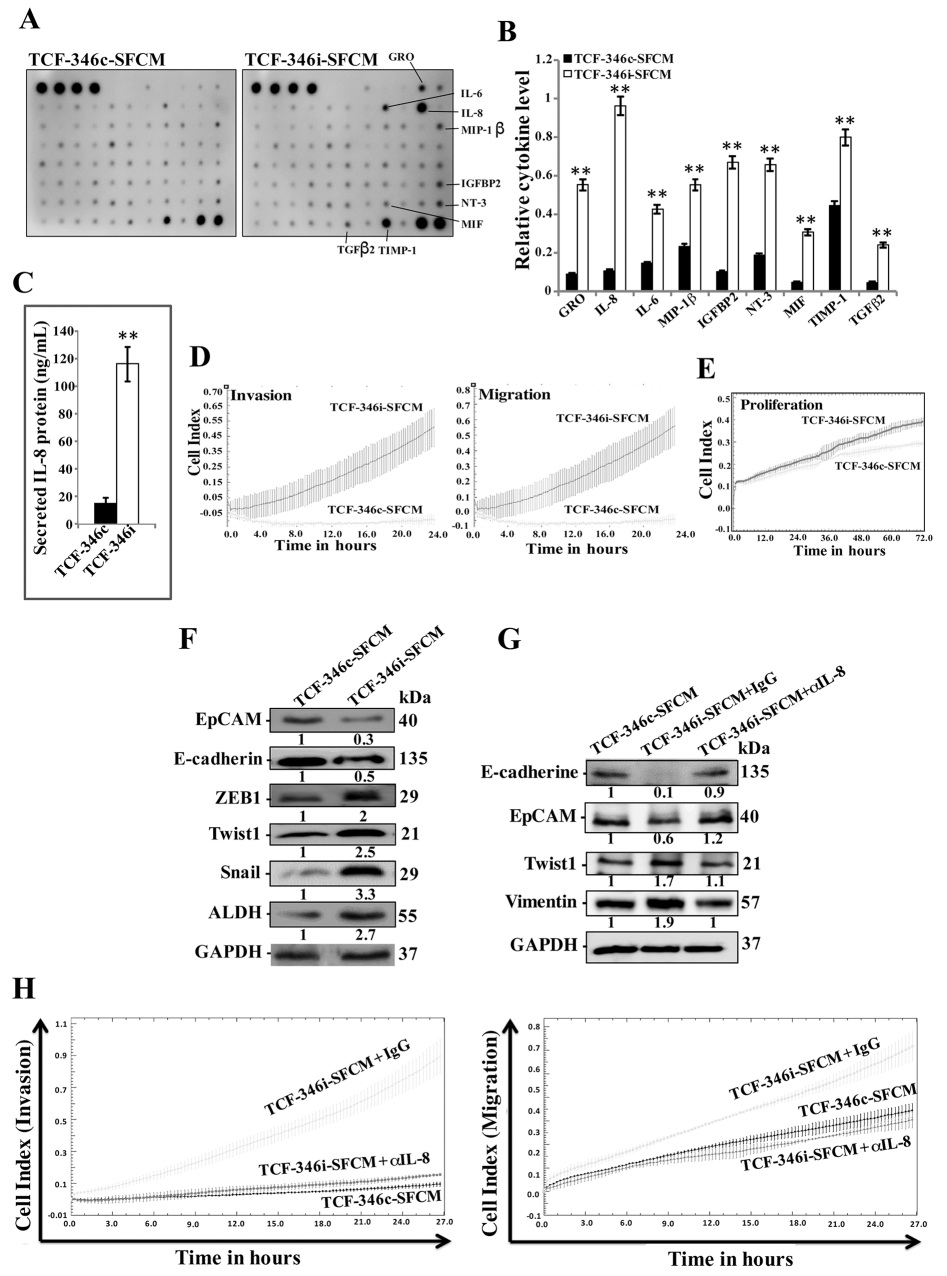
Let-7b inhibition triggers EMT in breast cancer cells in a paracrine manner **(A)** TCF-346i and TCF-346c cells were incubated for 24 h in SFM and the resulting SFCM (TCF-346i-SFCM and TCF-346c-SFCM, respectively) were applied to cytokine array membranes. **(B)** Histogram showing the expression levels of the indicated cytokines. The values were normalized against the 2 control spots (bottom right). Error bars indicate mean ± S.D, values of 2 independent experiments, ^**^P < 0.001. **(C)** ELISA for secreted IL-8. Error bars indicate mean ± S.D (n=3), ^*^P=0.045 and ^**^P=0.001. **(D** and **E)** MDA-MB-231 cells were seeded in the upper chambers of the CIM plates in presence of the indicated media, while the bottom chambers contained SFM. For proliferation, cells were seeded in the E-plates in the presence of the indicated SFCM. The migration/invasion and proliferation abilities were assessed by the real time RTCA-DP xCELLigence System. Data are representative of different experiments performed in triplicate. **(F)** Whole cell lysates were prepared from MDA-MB-231 cells treated as indicated and were used for immunoblotting using antibodies against the indicated proteins. **(G)** MDA-MB-231 cells were incubated in TCF-346c-SFCM or TCF-346i-SFCM media containing either IgG (TCF-346i-SFCM + IgG) or neutralizing anti-IL-8 antibody (0.5 μg/mL) (TCF-346i-SFCM + αIL-8), and then whole cell lysates were prepared and were used for immunoblotting using antibodies against the indicated proteins. The numbers under the bands represent fold changes relative to GAPDH. **(H)** MDA-MB-231 cells were seeded in the upper chambers of the CIM plates in presence of the indicated media, while the bottom chambers contained SFM, and migration and invasion abilities were assessed by the real time RTCA-DP xCELLigence System. Data are representative of different experiments performed in triplicate.

To further show the permanent active status of TCF-346i cells, we sought to test their paracrine effects. To perform this, serum-free medium (SFM) was conditioned with TCF-346c and TCF-346i cells for 24 h, and then the resulting serum-free conditioned media (SFCM) (TCF-346c-SFCM and TCF-346i-SFCM, respectively) were first used to assess the effect of let-7b knock-down on the secretion of various cytokines using the Ray Bio® human cytokine antibody arrays. Great differential levels of various cytokines were observed between TCF-346c and TCF-346i cells, including, GRO, IL-6, IL-8 and IGFBP2 (Figure 3A, 3B). These results were confirmed by ELISA for the most prominent protein IL-8. Indeed, the secreted level of this pro-carcinogenic cytokine was significantly increased in TCF-346i cells compared to controls (Figure 3B).

To test the paracrine effects of TCF-346i cells on breast cancer cells, MDA-MB-231 cells were seeded on the upper chamber wells of the CIM plates 16 with SFM, while the lower chamber wells contained TCF-346c-SFCM or TCF-346i-SFCM. The migration and invasion abilities of MDA-MB-231 cells were assessed using the RTCA-DP xCELLigence System. Figure 3C shows increase in the migration and invasion capacities of MDA-MB-231 cells treated with TCF-346i-SFCM as compared to those treated with TCF-346c-SFCM. Similarly, TCF-346i-SFCM enhanced the proliferation rate of MDA-MB-231 cells as compared to controls (Figure 3D).

In addition, we tested the effect of TCF-346c-SFCM and TCF-346i-SFCM on the epithelial-to-mesenchymal transition (EMT) markers in MDA-MB-231 cells by immunoblotting. Figure 3E shows that TCF-346i-SFCM down-regulated both epithelial markers E-cadherin and EpCam, while it increased the level of the mesenchymal markers ZEB1, Twist1 and Snail relative to cells exposed to TCF-346c-SFCM. Similarly, the stemness marker ALDH was also up-regulated (Figure 3E). This shows that TCF-346i cells enhanced the EMT process in MDA-MB-231 cells in a paracrine manner, which further confirms the active status of let-7b-deficient breast stromal fibroblasts.

Since let-7b inhibition strongly increased the expression/secretion of IL-8, we hypothesized the possible implication of this cytokine in the paracrine pro-EMT effects of TCF-346i cells. To verify this, we neutralized IL-8 in TCF-346i-SFCM using specific antibody, while IgG was utilized as control, and then MDA-MB-231 cells were treated as described above. Figure 3F shows that neutralizing IL-8 inhibited the pro-EMT process of TCF-346i-SFCM relative to cells treated with TCF-346i-SFCM containing IgG. Similarly, adding the neutralizing anti-IL-8 antibody to TCF-346i-SFCM inhibited its pro-migratory and -invasive effects as compared to cells exposed to TCF-346i-SFCM containing IgG, reaching a level similar to that obtained with TCF-346c-SFCM (Figure 3G). This indicates that the paracrine pro-carcinogenic effects of let-7b-deficient BSF are IL-8-related.

### Let-7b deficient fibroblasts stimulate breast cancer orthotopic tumor growth in mice

**Figure 4:**
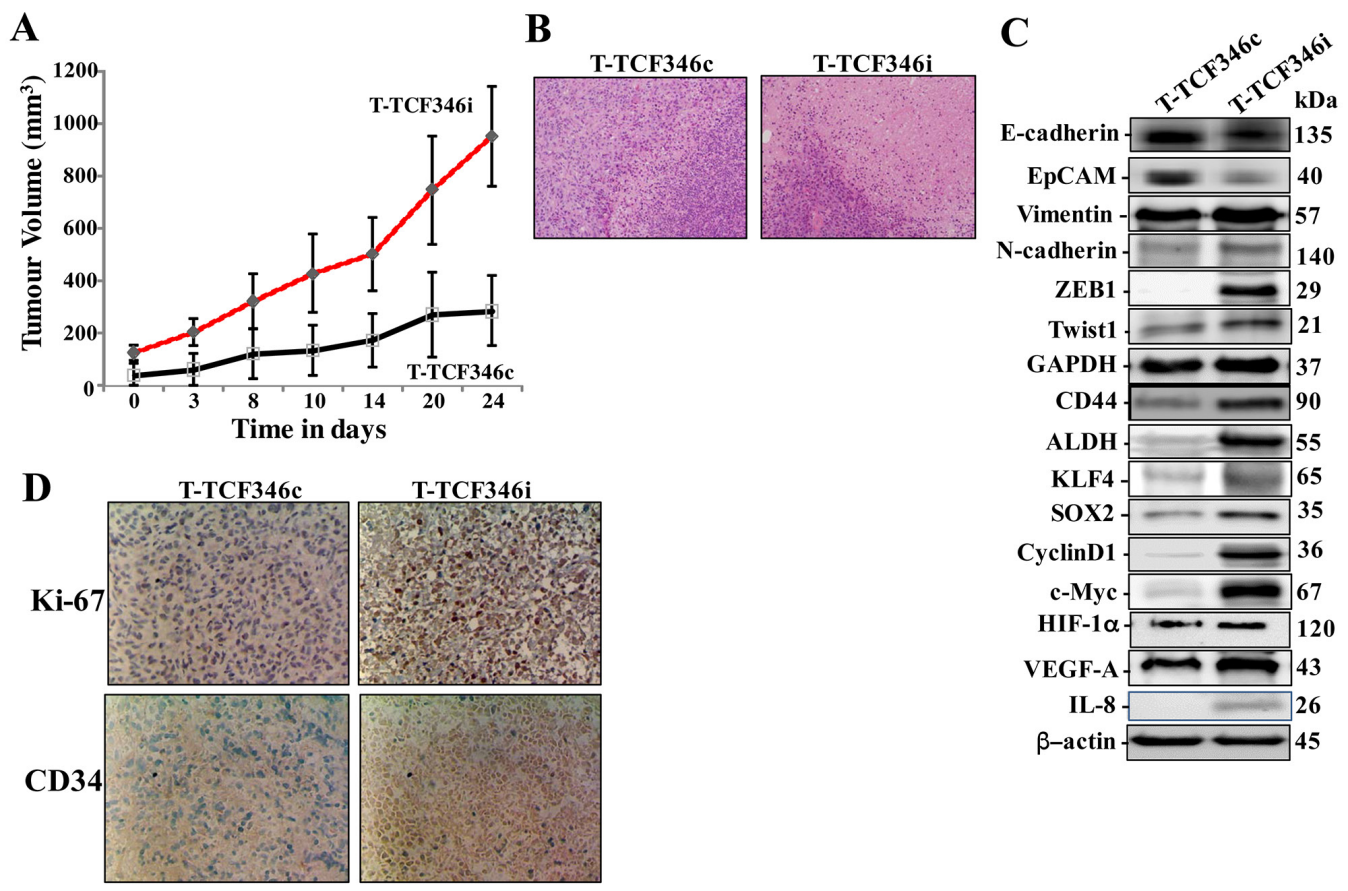
Let-7b-deficient breast stromal fibroblasts enhance orthotopic tumor growth Orthotopic breast cancer xenografts were created by co-injecting MDA-MB-231 cells with TCF-346c or TCF-346i cells under the nipple of the 4^th^ left fat pad of nude mice. **(A)** The histogram shows the volumes of xenograft tumors that were measured at the indicated time points. **(B)** Tissues were subjected to hematoxylin and eosin staining (Envision 40x). **(C)** Orthotopic tumors bearing TCF-346c cells (T-TCF-346c) or TCF-346i cells (T-TCF-346i) were excised and whole cell lysates were prepared and protein levels were assessed by immunoblotting using antibodies against the indicated proteins. **(D)** Excised tissues were subjected to immunohistochemistry against the indicated proteins.

To investigate the effect of let-7b inhibition in human breast fibroblasts on tumor growth *in vivo*, orthotopic breast tumor xenografts were created under the nipples the 4^th^ left fat pad of nude mice by co-implantation of MDA-MB-231 cells (2x10^6^) with either TCF-346i or TCF-346c cells (2x10^6^), which were passaged 4 times post-transfection. Tumors bearing TCF-346i fibroblasts (T-TCF346i) appeared earlier and grew much faster relative to those having control cells (T-TCF346c) (Figure 4A). This indicates that let-7b-deficient BSFs can enhance orthotopic tumor growth in mice. Interestingly, hematoxylin and eosin staining showed more necrosis in T-TCF346i as compared to T-TCF346c (Figure 4B). This shows that let-7b-deficient BSFs enhance tumor aggressiveness.

Additionally, whole cell extracts were prepared from T-TCF346i and T-TCF346i tissues, and the levels of various cancer- and metastasis-promoting genes were assessed by immunoblotting. Figure 4C shows that T-TCF346i tumors express higher level of the mesenchymal markers N-cadherin, ZEB1, Twsit1 and vimentin, while E-cadherin and EpCAM levels were lower as compared to T-TCF346c tumors. This confirms the *in vitro* results showing the enhancement of the EMT process by let-7b-deficient fibroblasts. In addition, TCF346i cells increased the level of the stemness markers CD44 and ALDH and the pluripotency markers KLF4 and SOX-2 relative to TCF346c cells (Figure 4C). Figure 4C also shows strong increase in the expression levels of the downstream proliferation effectors cyclin D1 and C-Myc in T-TCF346i tumors relative to controls. This was confirmed by immunohistochemistry showing higher Ki-67 staining in T-TCF346i tissue relative to controls (Figure 4D). This shows that the presence of let-7b-deficient breast stromal fibroblasts enhanced the growth as well as the EMT/stemness processes in breast cancer cells in orthotopic xenografts. In addition, let-7b-deficient BSFs enhanced the formation of new blood vessels in orthotopic tumors as shown by the strong increase in the staining of the endothelial cells marker CD34 in T-TCF346i as compared to T-TCF346c (Figure 4D). This indicates that let-7b-deficient BSFs promote angiogenesis in a paracrine manner. This could result from increase in the secretion of pro-angiogenic factors. Indeed, the levels of IL-8, HIF-1α and its downstream target VEGF-A were higher in T-TCF346i as compared to T-TCF346c, which explains the strong increase in the neo-vascularization shown in the tumors bearing TCF346i fibroblasts (Figure 4C, 4D).

### Let-7b mimic stably inhibits the STAT3/NF- κB/let-7b autocrine positive feedback loop and represses active breast fibroblasts

**Figure 5:**
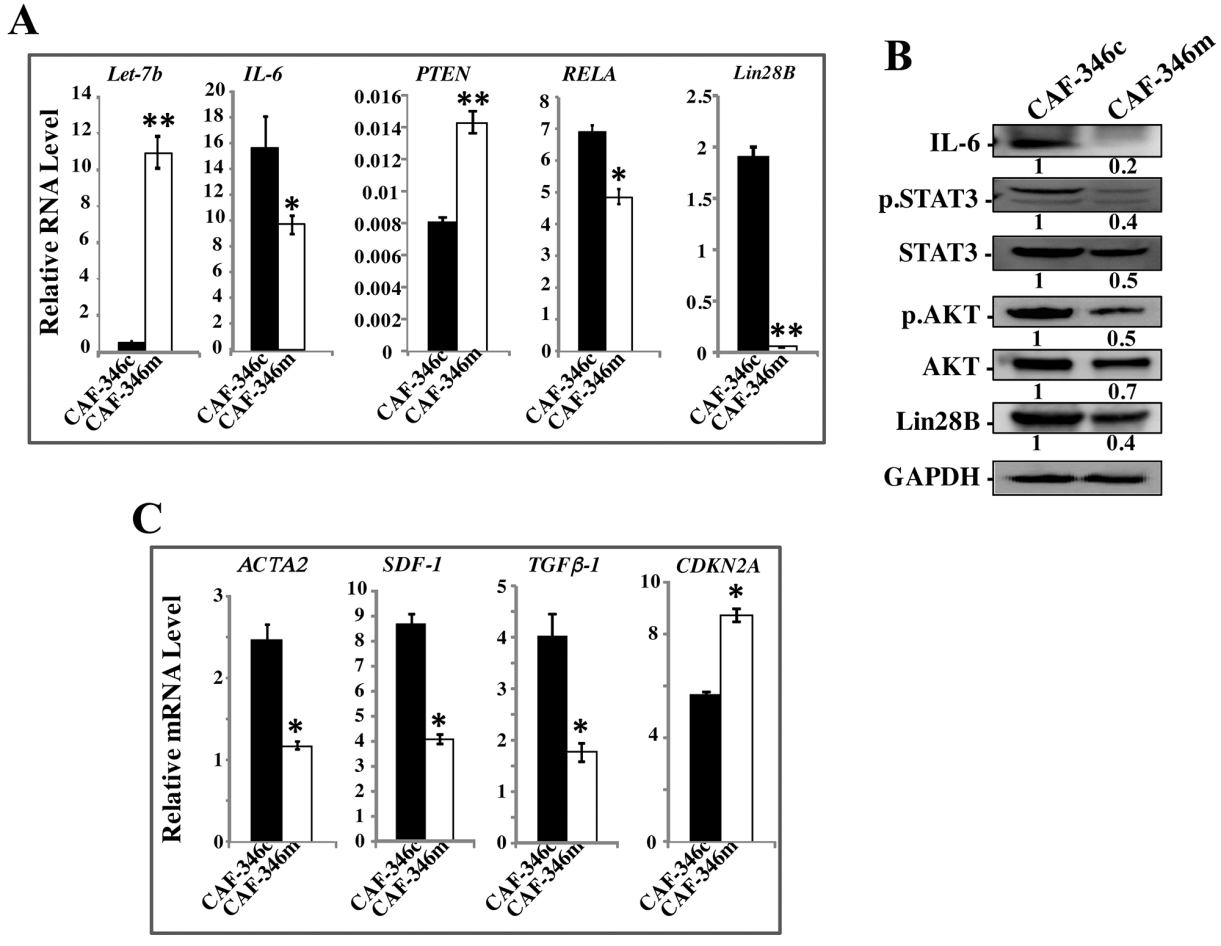
Increase in the level of let-7b inactivates the STAT3/NF-κB/let-7b positive feedback loop and suppresses myofibroblasts CAF-346 cells were transfected with let-7b mimic (CAF-346m) or a control sequence (CAF-346c) for 48 h, and then the medium-containing let-7b mimic was removed and replaced with let-7b mimic-free medium for 24 h. **(A)** and **(C)** Total RNA was purified and used for qRT-PCR. Experiments were performed in triplicate; error bars represent means ± S.D, values of 3 independent experiments (^*^P < 0.05 and ^**^P<0.001). **(B)** Cell lysates were prepared from the indicated cells and used for immunoblotting analysis using antibodies against the indicated proteins. The numbers under the bands represent fold changes relative to GAPDH. For the phosphorylated proteins, the levels were determined relative to their respective non-phosphorylated forms.

**Figure 6:**
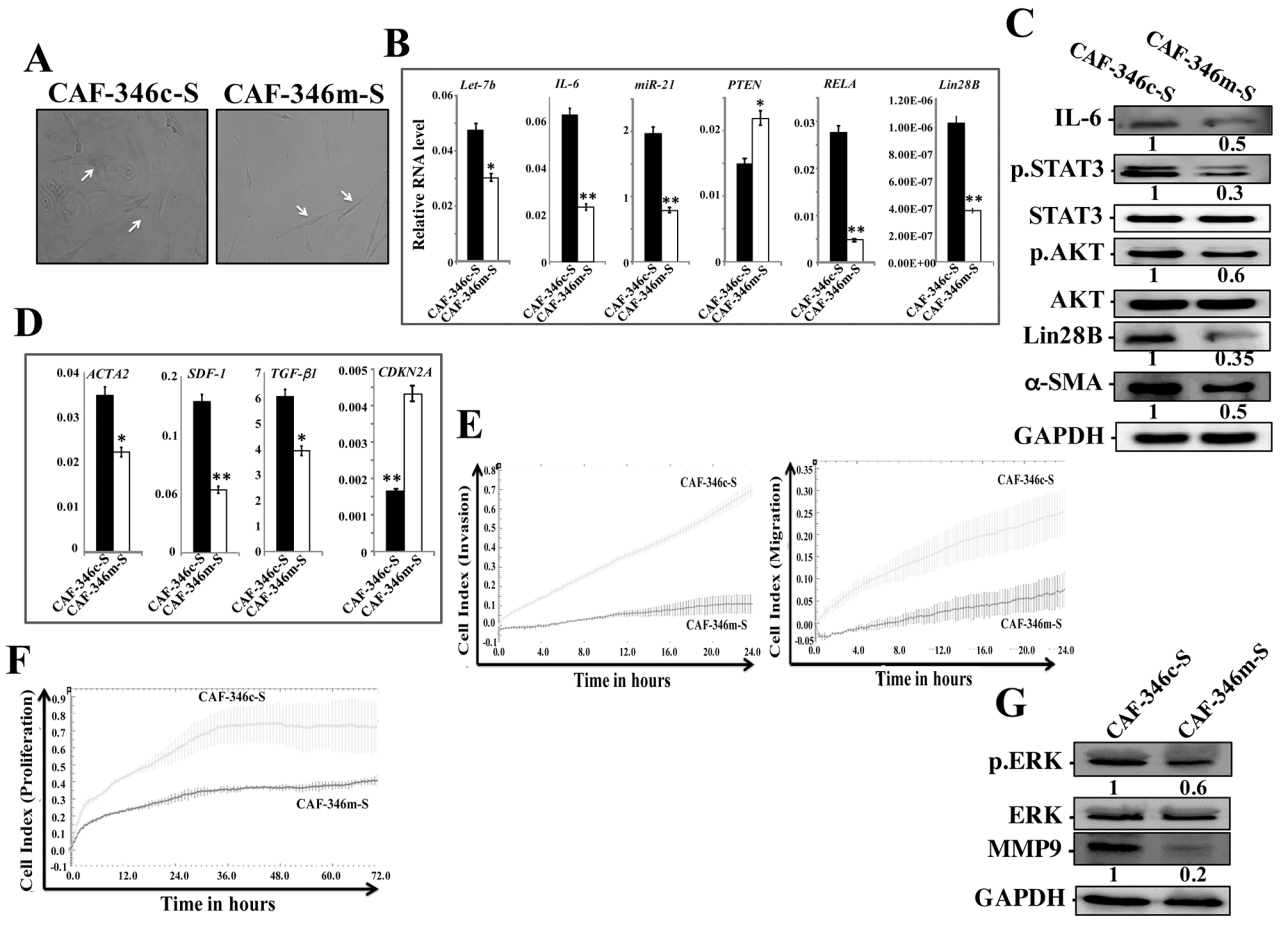
Increase in the level of let-7b persistently inactivates breast myofibroblasts and the STAT3/NF-κB/let-7b positive feedback loop CAF-346 cells were transfected with let-7b mimic (CAF-346m) or a control sequence (CAF-346c) for 48 h and then the medium-containing let-7b mimic was removed and replaced with let-7b mimic-free medium for 24 h, and then cells were splited and re-incubated in mimic-free medium 3 times. **(A)** Cell morphology in monolayer growth using phase contrast microscopy. **(B and D)** Total RNA was purified from the indicated cells and used for qRT-PCR. Experiments were performed in triplicate; error bars represent means ± S.D, values of 3 independent experiments (^*^P < 0.05 and ^**^P<0.001). **(C and G)** Whole cell lysates were prepared from the indicated cells and used for immunoblotting analysis using antibodies against the indicated proteins, and GAPDH was utilized as internal control. The numbers under the bands represent fold changes relative to GAPDH. For the phosphorylated proteins, the levels were determined relative to their respective non-phosphorylated forms. **(E)** Exponentially growing cells (10^4^) were added independently into the upper chambers of the 16-well CIM-plates, while the bottom chambers contained complete medium. **(F)** cells were seeded in the E-plates in the presence of complete medium, and cell proliferation was assessed for 72 h. Cell invasion/migration and proliferation were assessed using the RTCA-DP xCELLigence System. Data are representative of different experiments performed in triplicate.

To further confirm the role of let-7b in the activation of breast stromal fibroblasts, we investigated the effect of increased level of let-7b on active BSFs. Therefore, CAF-346 and CAF-64 cells were transfected with let-7b mimic (CAF-346m and CAF-64m, respectively) or a control sequence (CAF-346c and CAF-64c, respectively) for 24 h, and then total RNA was purified and the level of the let-7b microRNA was assessed. Figures 5A and Supplementary Figure 3A show highly significant increase in the level of let-7b in CAF-346m and CAF-64m as compared to their respective controls. Expectedly, the mRNA level of the let-7b downstream target *IL-6* was significantly decreased in CAF-346m relative to controls (Figure 5A). Similar effect was observed for miR-21, *RELA* and lin28B, whilst *PTEN* was up-regulated in CAF-346m as compared to the control cells (Figure 5A). Furthermore, let-7b mimic reduced the protein levels of IL-6, Lin28B, active STAT3 (p-STAT3) as well as active AKT (p-AKT) (Figure 5B). This indicates that the increase in the amount of let-7b inhibited the IL-6-dependent positive feedback loop. Concomitantly, the increase in the level of let-7b decreased the mRNA levels of the 3 main myofibroblast markers (*ACTA2*, *SDF-1* and *TGF-β1*), while up-regulated *CDKN2A* in CAF-346m as compared to control cells (Figure 5C). This indicates that the increase in the amount of let-7b inactivated myofibroblats through inhibiting the STAT3/NF-κB/let-7b autocrine positive feedback loop. Indeed, let-7b mimic transformed the shape of cells from large and flat (characteristic of myofibroblasts) to rounded spindle-shaped, which characterize normal fibroblasts (Figure 6A). To test the persistent effect of let-7b mimic, cells were passaged several times, and then the expression of different relevant genes was assessed. Importantly, let-7b level was reduced in both CAF-346m-S and CAF-64m-S relative to their respective control cells (Figure 6B and Supplementary Figure 3B). On the other hand, IL-6, miR-21, RELA and lin28B remained down-regulated while PTEN remained up-regulated in CAF-346m-S and CAF-64m-S cells relative to controls (Figure 6B and Supplementary Figure 3B). At the protein levels, the IL-6 and Lin28B levels remained reduced, while STAT3 and AKT remained inactive (Figure 6C). Moreover, the level of α*-SMA* remained reduced at both the mRNA and protein levels (Figure 6C and 6D). The lower levels of *SDF-1* and *TGF* β*-1* were also sustained, while the *CDKN2A* mRNA levels remained up-regulated in CAF-346m-S and CAF-64m-S as compared to their respective control cells (Figure 6D and Supplementary Figure 3C). This was confirmed by showing reduced migratory, invasive and proliferative capacities of CAF-346m-S cells relative to controls (Figure 6E and 6F). These cellular results were corroborated with decrease in the levels of p-ERK1/2 and MMP-9 in CAF-346m-S as compared to CAF-346c-S cells (Figure 6G). These results indicate that ectopic increase in let-7b level stably reverted myofibroblasts to inactive fibroblasts through inactivation of the STAT3/NF-κB/let-7b feedback loop.

**Figure 7:**
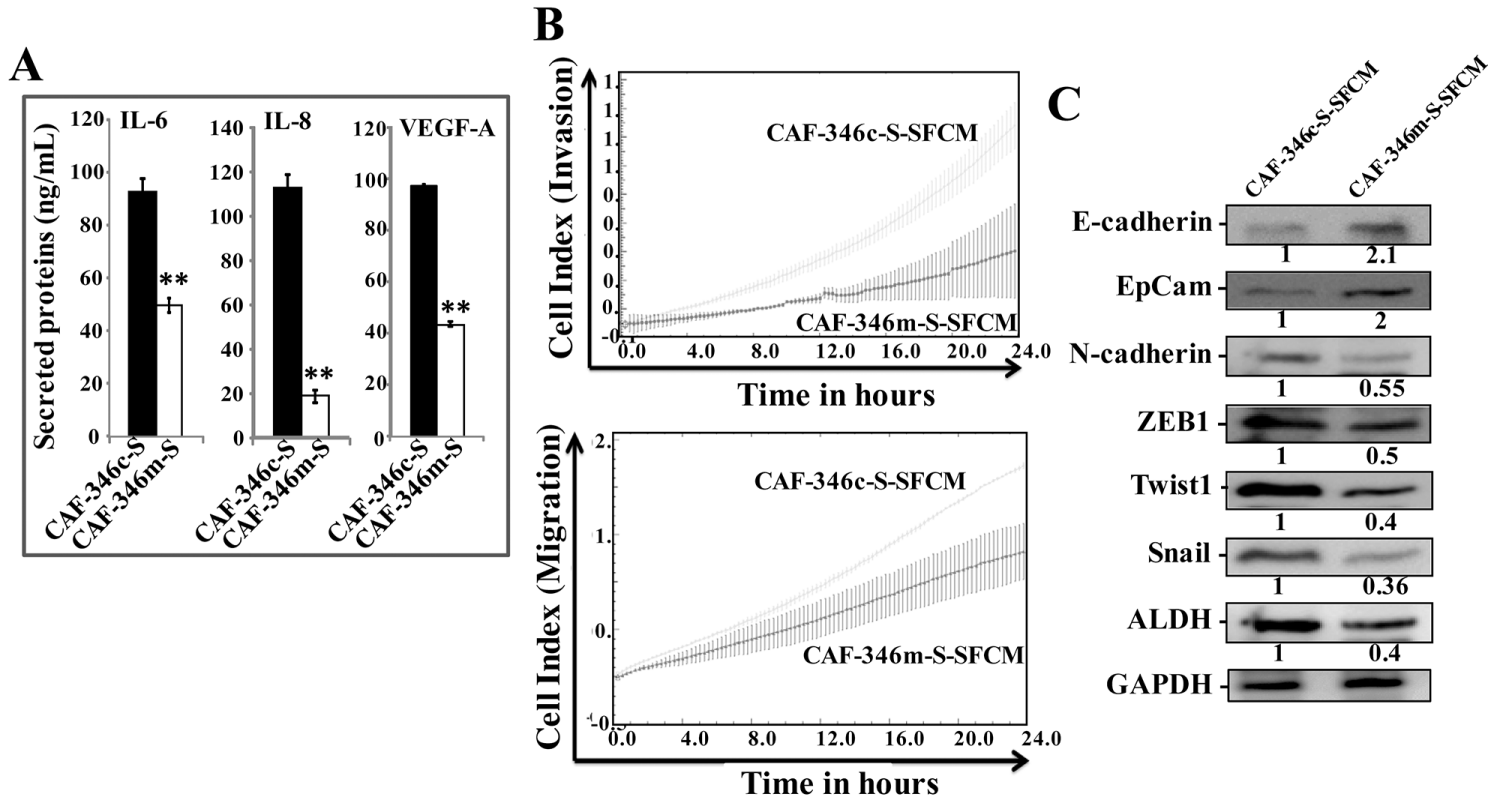
Overexpression of let-7b by mimic decreased cancer-promoting abilities of breast myofibroblasts **(A)** SFCM from the indicated cells were collected, and the levels of the indicated proteins were determined by ELISA and were presented in the respective histograms. Error bars indicate mean ± S.D. ^**^, *P*<0.001 (n=3). **(B)** MDA-MB-231 cells were seeded in the presence of the indicated SFCM as described in Figure 2D, and then the migration and invasion abilities were assessed by the real time RTCA-DP xCELLigence System. Data are representative of different experiments performed in triplicate. **(C)** Whole cell lysates were prepared from MDA-MB-231 cells treated as indicated and were used for immunoblotting using antibodies against the indicated proteins. The numbers under the bands represent fold changes relative to GAPDH.

Furthermore, ELISA indicates significant decrease in the secretion of IL-6, IL-8 and VEGF-A from CAF-346m-S relative to controls (Figure 7A). This prompted us to investigate the paracrine effect of CAF-346m-S cells and their corresponding controls on breast cancer MDA-MB-231 cells. To perform this, SFM were conditioned with CAF-346c-S and CAF-346m-S cells for 24 h, and then the resulting SFCM were added to MDA-MB-231 cells. Figure 7B shows decrease in the invasive/migratory capacities of MDA-MB-231 cells treated with CAF-346m-S.SFCM as compared to those treated with CAF-346c-SFCM. Furthermore, CAF-346m-S.SFCM up-regulated the epithelial makers (E-cadherin and EpCAM), whilst it reduced the levels of the mesenchymal markers (N-cadherin, ZEB1, Twist1 and Snail) (Figure 7C). This suggests that CAF-346m-S.SFCM inhibits the EMT process in MDA-MB-231 breast cancer cells. Likewise, CAF-346m-S.SFCM reduced the expression level of the stemness marker ALDH (Figure 7C). Together, these results indicate that let-7b mimic represses the procarcinogenic effects of active breast stromal fibroblasts.

### IL-8 is under the control of let-7b and is highly expressed in CAF cells

**Figure 8:**
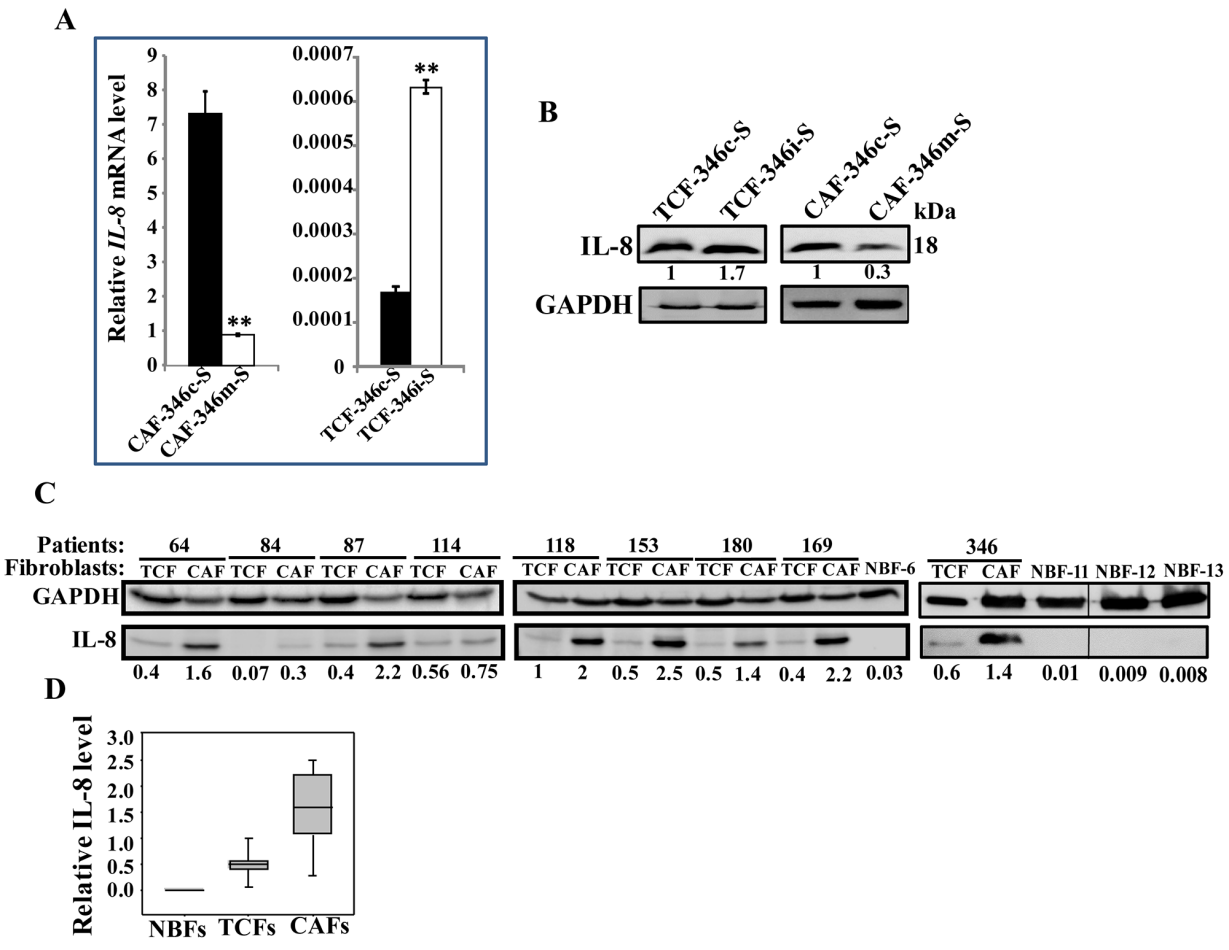
Let-7b negatively controls IL-8, which is highly expressed in CAF cells **(A)** Total RNA was purified from the indicated cells, and IL-8 level was assessed by qRT-PCR using specific primers. Experiments were performed in triplicate and several times; error bars represent means ± S.D, values of 2 independent experiments (^**^,*P* < 0.001). **(B and C)** Whole cell lysates were prepared from the indicated cells and were used for immunoblotting using antibodies against the indicated proteins. The numbers under the bands represent fold changes relative to GAPDH. **(D)** Box plot showing IL-8 expression levels in the indicated types of breast fibroblasts as determined in Figure 8C.

Based on the results obtained in Figure 3, we sought to investigate the possible implication of let-7b in the control of IL-8 expression. Therefore, the IL-8 mRNA and protein levels were assessed in CAF-346m, TCF-346i and their respective control cells CAF-346c and TCF-346c. Figure 8A shows that while let-7b mimic significantly reduced the level of the IL-8 mRNA, let-7b inhibition strongly up-regulated IL-8 as compared to their respective controls. This let-7b-dependent expression of the IL-8 mRNA was also observed at the protein level (Figure 8B). This indicates that let-7b negatively controls the expression of IL-8. Thereby, we assessed the IL-8 level in various CAF/TCF pairs derived from different breast cancer patients and also normal breast fibroblasts (NBFs) derived from plastic surgery. Figure 8C shows that the IL-8 expression level was higher in 8 out of 9 (89%) CAFs relative to their corresponding TCFs, and in all TCF cells as compared to NBFs. Indeed, IL-8 was almost undetectable in NBFs, while CAFs exhibited the highest levels (Figure 8D). This mirrors let-7b down-regulation in the majority of CAF cells compared to their TCF counterparts (Figure 1A).

## DISCUSSION

Accumulating evidence indicates that microRNAs play major roles in both the onset as well as the progression of various types of cancer, including breast carcinomas [12–14]. These effects are not only autocrine, but also paracrine and through secreted exosomes. Indeed, these small non-coding RNAs are both mediators and communicators between cancer cells and their microenvironment [15]. MicroRNAs play also important roles in modulating the biology of cancer-associated fibroblasts towards a more permissive pro-tumoral phenotype [16–18]. In the present report, we have shown that the tumor suppressor microRNA let-7b is down-regulated in most breast cancer-associated fibroblasts compared to their adjacent paired TCFs; and specific let-7b inhibition granted BSFs with traits of activated fibroblasts, including enhanced migration/invasion and proliferation. Like in cancer cells, this effect was persistent through the activation of the IL-6/STAT3/NF-κB positive feedback loop, which is responsible for the persistent activation of BSFs [4]. The activation of this loop may also provide a molecular explanation for let-7b down-regulation in CAFs. Indeed, let-7b inhibitor Lin28B, is up-regulated in response to the induction of the IL-6 feedback loop in both cancer and fibroblast cells [4, 19]. Previously, Zhao et al have identified several dysregulated miRNAs in breast CAFs, and miR-200 family members were severely suppressed. Subsequently, they have shown that downregulation of miR200s activated breast stromal fibroblasts [20, 21]. Similarly, it has been shown that miR-26b is down-regulated in CAFs from ER-positive breast cancers, and when specifically down-regulated it activated normal breast fibroblasts [22]. This indicates that several miRNAs, like the important tumor suppressor genes p16, p53 and PTEN, also suppress breast stromal fibroblasts, may be through repressing their target onco/inflammatory genes [23, 24]. Several publications have reported the role of microRNAs in activating stromal fibroblasts through modulating the expression of important inflammatory cytokines, such as IL-6 and CCL5 [25, 26]. Likewise, we have shown here that let-7b also controls the secretion of several important cytokines such as IL-6, IL-8, Gro, Gro-α and others (Figure 3A). This prompted us to investigate the paracrine effects of let-7b-deficient BSFs. Importantly, let-7b inhibition enhanced the pro-carcinogenic effects of BSFs in a paracrine manner both *in vitro* and in orthotopic tumor xenografts. These extracellular effects of let-7b-deficient cells are IL-8-dependent. Indeed, these cells secreted high levels of IL-8, and specific inhibition of this cytokine in the corresponding SFCM suppressed the paracrine induction of the EMT process in breast cancer cells (Figure 3). In fact, let-7b represses the expression of IL-8 at both the mRNA and protein levels. Since IL-8 is not a target of let-7b and its 3’UTR does not contain specific let-7b seeding site, this let-7b-dependent regulation of IL-8 expression could be indirect through the IL-6/STAT3/NF-κB positive feed back loop. It is well known that IL-8 is a target of the transcription factors STAT3 and NF-κB [27]. Similarly, the expression of IL-8 was inhibited in *H.pylori* infected gastric epithelial cells with let-7b overexpression and the consequent NF-κB down-regulation [28]. This shows that let-7b negatively controls 2 major imflammatory/cancer-related cytokines IL-6 and IL-8. This was confirmed by showing that most CAFs express high level of IL-8 as compared to their corresponding TCFs. Additionally, we have shown that TCFs, which were generated from histologically normal breast tissues, exhibited higher levels of IL-8 relative to those observed in normal breast fibroblasts. This confirms that TCFs have some active features as previously shown, and thereby may play important roles in local recurrence [29].

Interestingly, inhibition of let-7b in BSF promoted breast cancer tumor growth, EMT, stemness and angiogenesis in orthotopic humanized tumors, which indicates that let-7b has non-cell-autonomous tumor suppressive functions. These could be mediated through several cancer-promoting factors such as IL-6 and IL-8.

These results were strengthened through overexpression of let-7b by mimic in active CAF cells wherein phenotypes of normal breast fibroblasts were restored. Indeed, CAF cells became fusiform, they expressed lower levels of α-SMA, SDF-1, TGF-β1 and IL-8, they were less migratory/invasive and proliferative, with strong reduction in their paracrine pro-carcinogenic effects. These data further confirmed the tumor suppressive function of let-7b. Likewise, ectopic expression of miR-200s in CAFs partially restored normal features of breast fibroblasts [20]. Similarly, re-expression of miR-31 in CAFs inhibited their capacity to promote endometrial tumor cell migration and invasion [30]. This conversion from active CAFs to normal fibroblasts by miRNAs was also observed in ovarian CAFs [25]. This indicates that several miRNAs are key players in suppressing the pro-carcinogenic effects of breast stromal fibroblasts through post-transcriptional gene regulation.

In summary, the present findings indicate that let-7b down-regulation is an important step toward the activation of breast stromal fibroblasts. On the other hand, increase in the let-7b level by mimic normalizes active breast myofibroblasts, and consequently can be utilized to enhance the efficiency of the classical anti-cancer regimen.

## MATERIALS AND METHODS

### Cells, cell culture and chemicals

Breast fibroblast cells were obtained and used as previously described [31]. MDA-MB-231 cells were obtained from ATCC and were authenticated at ATCC before purchase by their standard short tandem repeat DNA typing methodology, and were routinely tested for the presence of the relevant markers, and were cultured following the instructions of the company. All supplements were obtained from Sigma (Saint Louis, MO, USA) except for antibiotic and antimycotic solutions, which were obtained from Gibco (Grand Island, NY, USA). The neutralizing anti-IL-8 monoclonal antibody (clone # 6217) was purchased from R&D Systems, USA.

### Cellular lysate preparation and immunoblotting

This has been performed as previously described [32]. Antibodies directed against alpha smooth muscle actin (α-SMA), Twist-1, Vimentin (RV202), N-cadherin, transforming growth factor beta 1 (TGF-β1), VEGF-A (ab46154) and IL-6 were purchased from Abcam (Cambridge, MA); Lin28B (D4H1), AKT (C73H10), p-AKT (Thr308), STAT3, pSTAT3-Tyr705 (D3A7), Snail (C15D3), E-cadherin (24E10), EpCam (UV1D9), JAK-2 (D2E12), phospho-JAK-2 (TYR1007/1008), Cyclin D1 (2922), Akt, phospho-Akt (193H12), NF-κB, ERK1/2 (137F5), phospho-ERK1/2, MMP-9, HIF-1α, Glyceraldehydes-3-phosphate dehydrogenase (GAPDH, 14C10) and β-Actin from Cell Signaling (Danvers, MA); ZEB-1 (4C4) from Abnova (Taipei, Taiwan), ALDH1A1 from Sigma (CA, USA) and IL-8 (EpR1116(2)) from Origene (Rockville, MD, USA).

### RNA purification and qRT-PCR

Total RNA was purified using the Qiagen kit according to the manufacturer’s instructions, and was treated with RNase-free DNase before cDNA synthesis using the RT-PCR Kit (Clontech, USA) for mRNAs, while the Qiagen miScript II RT Kit was utilized for miRNAs. For qRT-PCR, the RT^2^ Real-Time^™^ SYBR Green qPCR mastermix (Roche, Germany) was used for mRNAs, while the Qiagen miScript Syber Green PCR Kit was used for miRNAs, and the amplifications were performed utilizing the light cycler 480 (Roche, Germany). The melting-curve data were collected to check PCR specificity, and the amount of PCR products was measured by threshold cycle (Ct) values and the relative ratio of specific genes to *GAPDH* (or U6 for mature miRNAs) for each sample was then calculated. The respective primers are:

**Table:** 

Primers	Forward	Reverse
*CDKN2A*	5’-GAGGCCGATCCAGGTCATGA-3’	5’-GCACGGGTCGGGTGAGA-3’
*GAPDH*	5’-GAGTCCACTGGCGTCTTC-3’	5’-GGGGTGCTAAGCAGTTGGT-3’
*SDF-1*	5’- CTCAACACTCCAAACTGTGCCC -3’	5’-CTCCAGGTACTCCTGAATCCAC-3’
*ACTA2(α-SMA)*	5′-CTATGCCTCTGGACGCACAACT -3′	5′-CAGATCCAGACGCATGATGGCA -3′
*TGF-β1*	5’-TACCTGAACCCGTGTTGCTCTC -3’	5’-GTTGCTGAGGTATCGCCAGGAA -3’
*IL-6*	5’-AGACAG CCA CTC ACC TCT TCA G -3’	5’- TTC TGC CAG TGC CTC TTT GCT G -3’
*RELA*	5’-CACGATGGTGACCTCCTTCT-3’	5’-GCAGGTGCGTTTCTATGACA-3’
*PTEN*	5’-GCGGAACTTGCAATCCTCAGT-3’	5’-GCTGAGGGAACTCAAAGTACTGAA-3’
*Lin28B*	5’-CCTGTTTAGGAAGTGAAAGAAGAC-3’	5’-CACTTCTTTGGCTGAGGAGGTAG-3’
*IL-8*	5’-GATCCACAAGTCCTTCCA-3’	5’-GCTTCCACATGTCCTCACAA-3’
Mature *Let-7b*	5’-TGAGGTAGTAGGTTGTGTGGTT-3’	
Mature *miR-21*	5’-TAGCTTATCAGACTGATGTTGA-3’	
*U6*	5’- CGCAAGGATGACACGCAAATTC-3’	

### ELISA assays

Supernatants from 24 h fibroblast cell cultures were harvested and ELISA was performed according to the manufacturer’s instructions (R&D Systems). The OD was used at 450 nm on a standard ELISA plate-reader. These experiments were performed in triplicates, and were repeated several times.

### Transfections

Let-7b inhibitor and its corresponding control as well as let-7b mimic and its corresponding control were obtained from Qiagen. The transfections were carried out using the RNA HiPerFect reagent (Qiagen) using 100 nM let-7b miRNA mimics or 100 nM let-7b miRNA inhibitor (Qiagen, USA) in 60-mm dishes according to the manufacturer’s instructions.

Anti-hsa-let-7b-5p miScript miRNA Inhibitor (MIN0000063) sequence:

CGGGGUGAGGUAGUAGGUUGUGUGGUUUCAGGGCAGUGAUGUUGCCCCUCGGAAGAUAACUAUACAACCUACUGCCUUCCCUG

Syn-hsa-let-7b-5p miScript miRNA Mimic (MSY0000063) sequence:

CGGGGUGAGGUAGUAGGUUGUGUGGUUUCAGGGCAGUGAUGUUGCCCCUCGGAAGAUAACUAUACAACCUACUGCCUUCCCUG.

### Cell migration, invasion and proliferation

These assays were performed in a real-time and label-free manner using the xCELLigence RTCA technology (Roche, Germany) that measures impedance changes in a meshwork of interdigitated gold microelectrodes located at the bottom well (E-plate) or at the bottom side of a micro-porous membrane (CIM plate 16). Cell migration and invasion were assessed as per manufacturer’s instructions. In brief, 2x10^4^ cells in serum-free medium were added to the upper wells of the CIM-plate with thin layer of matrigel basement membrane matrix (for invasion) or without (for migration), and a complete media was added to the lower chamber wells used as a chemo-attractant. Subsequently, the plates were incubated in the RTCA for 24 h and the impedance value of each well was automatically monitored by the xCELLigence system and expressed as a Cell index value which represents cell status based on the measured electrical impedance change divided by a background value. Each assay was performed in triplicate.

For the proliferation assay, exponentially growing cells (2×10^4^) were seeded in E-plate with complete medium as per manufacturer’s instruction. All data were recorded and analyzed by the RTCA software. Cell index (CI) was used to measure the change in the electrical impedance divided by the background value to represent cell status. Each assay was performed in triplicate.

### Immunohistochemistry staining on FFPE tissues

Immunohistochemistry on formalin-fixed paraffin-embedded tissues was performed using anti-Ki-67 and CD34 antibodies (Abcam) at dilutions of 1:100 and 1:150, respectively, and then slides were stained using automated staining platform (Ventana). Envision + polymer (ready to use; Dako) was used as a secondary antibody. Color was developed with 3,3′-diaminobenzidine (DAB) and instant hematoxylin (Shandon) was used for counterstaining.

### Orthotopic tumor xenografts

Animal experiments were approved by the KFSH&RC institutional Animal Care and Use Committee (ACUC) and were conducted according to relevant national and international guidelines. 10 female nude mice were randomized into 2 groups and breast cancer orthotopic xenografts were created by coimplantation of the MDA-MB-231 cells (2 × 10^6^) with TCF-346i or TCF-346c cells (2 × 10^6^) under the nipple of the 4^th^ left fat pad of each mouse. Tumour size was measured with a caliper using the following formula (Length X Width X Height).

### Conditioned media

Cells were cultured in medium without serum for 24 h, and then media were collected and briefly centrifuged. The resulting supernatants were used either immediately or were frozen at -80°C until needed.

### Statistical analysis

Statistical analysis was performed by student’s *t*-test and *P* values of 0.05 and less were considered as statistically significant.

### Quantifications

The expression levels of the immunoblotted proteins were measured using the Image J software. Protein signal intensity of each band was determined, and then dividing the obtained value of each band by the values of the corresponding internal control allowed a correction of the loading differences.

## SUPPLEMENTARY MATERIALS FIGURES


