# Supplementary material for: Let-7b inhibits cancer-promoting effects of breast cancer-associated fibroblasts through IL-8 repression
Source: Oncotarget. 2018 Apr 3;9(25):17825–38. doi: 10.18632/oncotarget.24895 (PMC5915157; doi:10.18632/oncotarget.24895)
Supplement: Supplementary file 1 [file oncotarget-09-17825-s001.pdf]

# Let-7b inhibits cancer-promoting effects of breast cancer-associated fibroblasts through IL-8 repression

## SUPPLEMENTARY MATERIALS

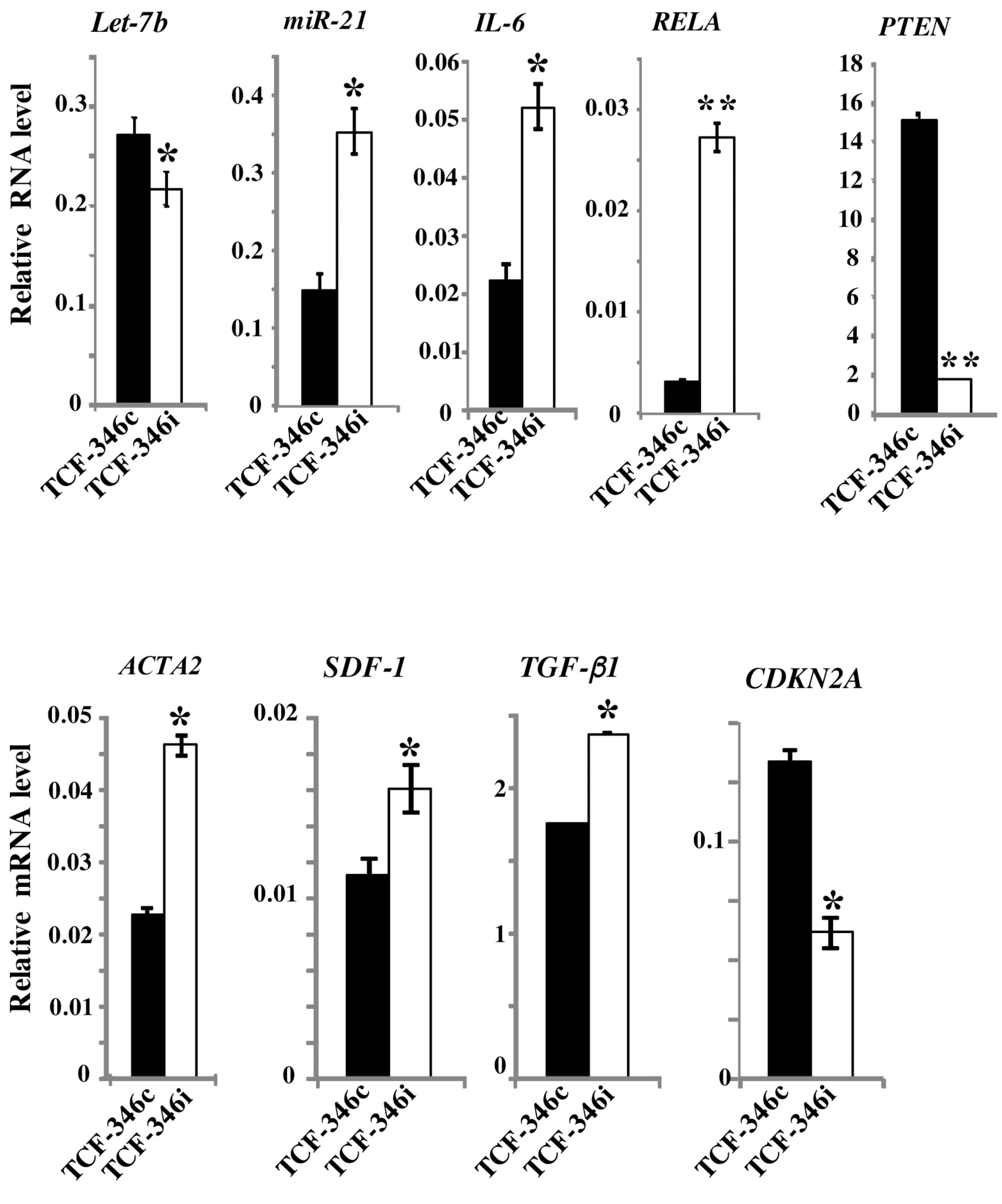


**Supplementary Figure 1: Let-7b inhibition activates breast stromal fibroblasts through induction of the STAT3/NF-κB/let-7b positive feedback loop.** TCF-346 cells were transfected with let-7b inhibitor (TCF-346i) or control (TCF-346c) for 48 h, medium was replaced with inhibitor-free medium for 24h, and then cells were passaged once. Total RNA was purified and used for qRT-PCR. Experiments were performed in triplicate and several times; error bars represent means ± S.D ($^{*}P < 0.05$ and $^{**}P<0.001$).

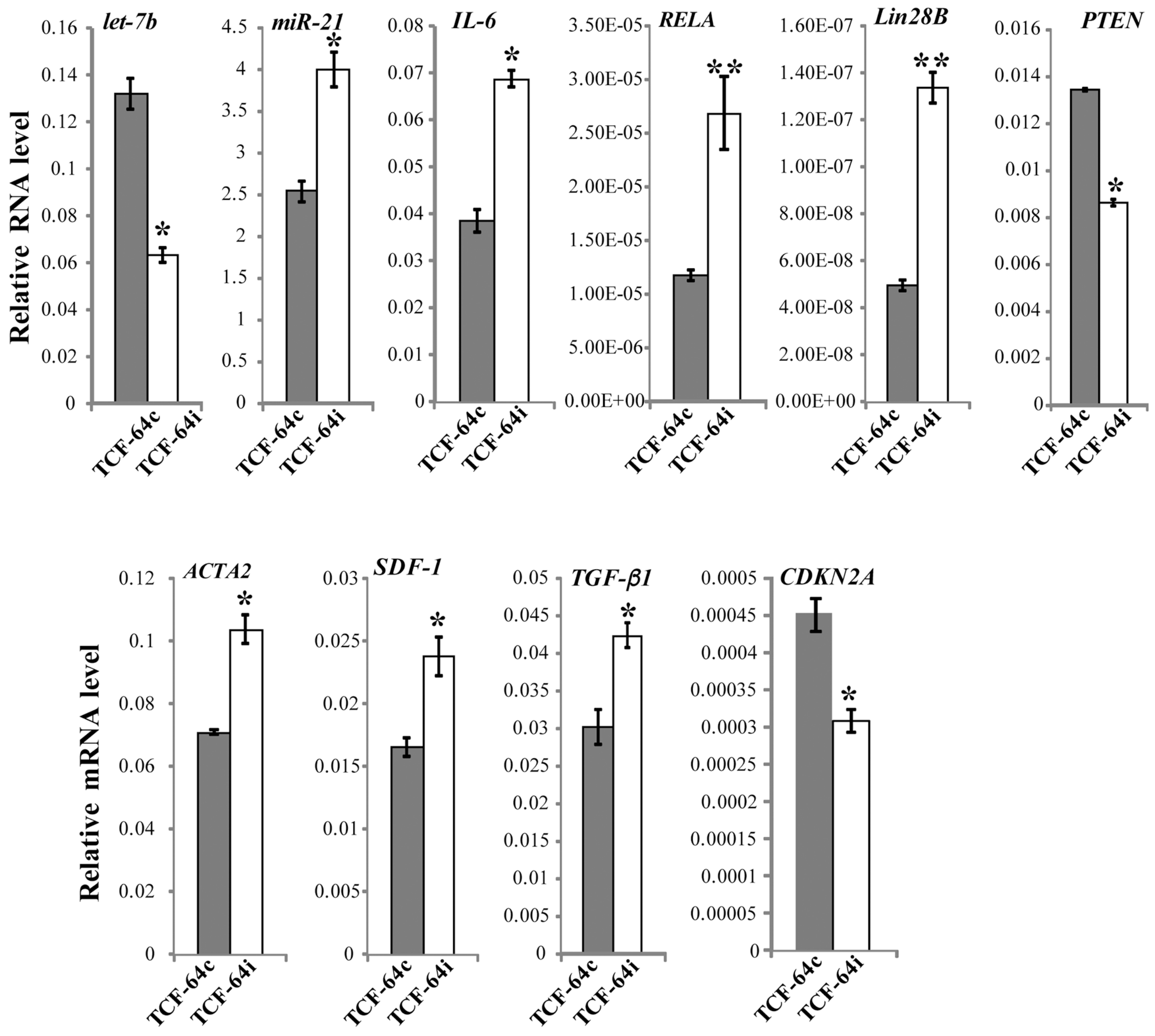


**Supplementary Figure 2: Let-7b inhibition persistently activates STAT3/NF-κB/let-7b positive feedback loop and breast stromal fibroblasts.** TCF-64 cells were transfected with let-7b inhibitor (TCF-64i) or control (TCF-64c) for 24 h, and then the medium-containing let-7b inhibitor was removed and replaced with let-7b inhibitor-free medium for 48 h, and then cells were passaged and re-incubated in inhibitor-free medium 4 times. Total RNA was purified from the indicated cells and used for qRT-PCR. Experiments were performed in triplicate and several times; error bars represent means ± S.D ($^{*}P < 0.05$ and $^{**}P<0.001$).

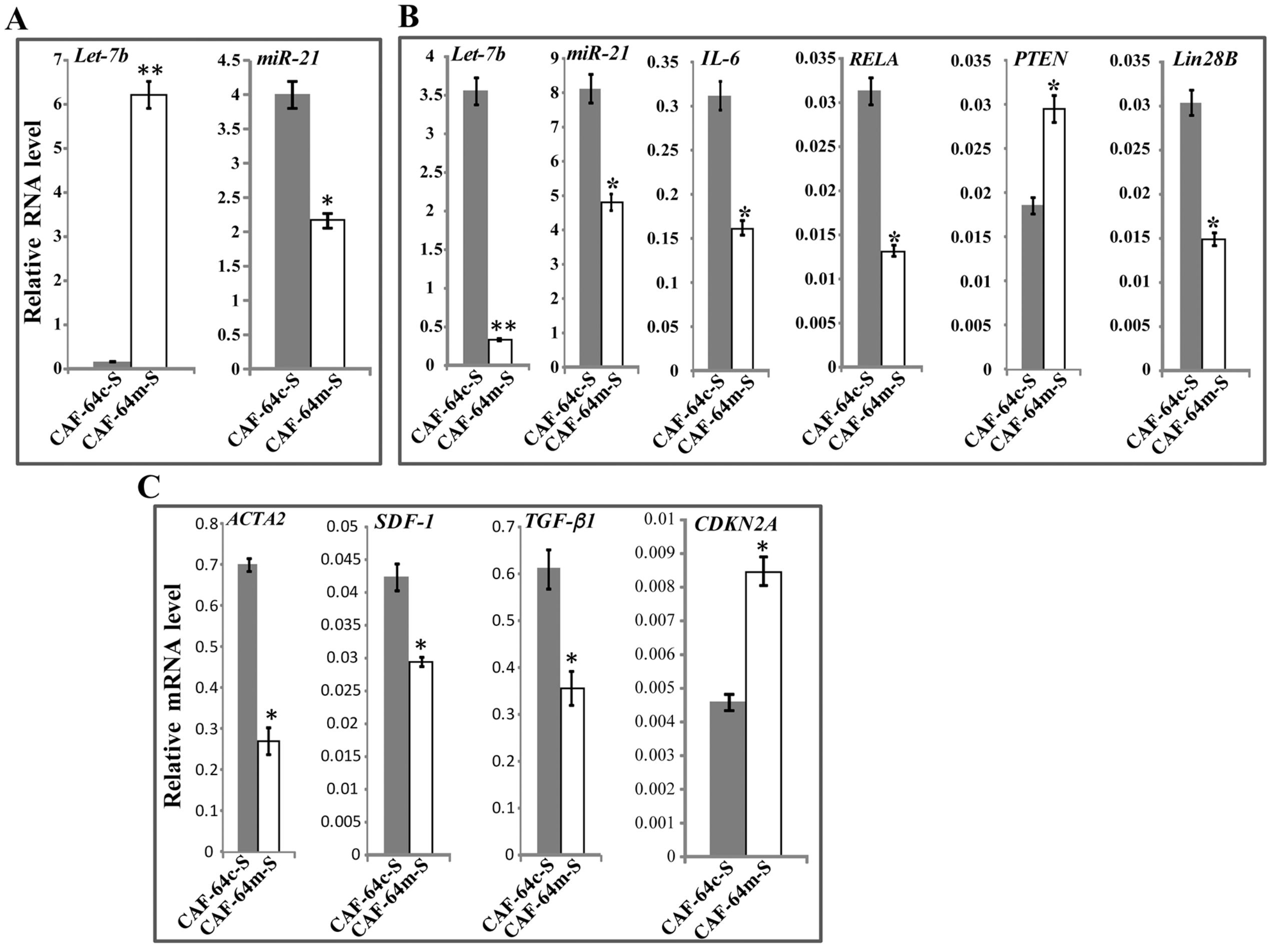


**Supplementary Figure 3: Increase in the level of let-7b persistently inactivates breast myofibroblasts and the STAT3/NF-κB/let-7b positive feedback loop.** **(A)** CAF-64 cells were transfected with let-7b mimic (CAF-64m) or a control sequence (CAF-64c) for 48 h, and then the medium-containing let-7b mimic was removed and replaced with let-7b mimic-free medium for 24 h. **(B and C)** CAF-64m and CAF-64c were passaged 4 times and generated CAF-64m-S and CAF-64c-S, respectively. Total RNA was purified and used for qRT-PCR. Experiments were performed in triplicate and several times; error bars represent means ± S.D ($^{*}P < 0.05$ and $^{**}P<0.001$).
